# Specific Metabolites Involved in Antioxidation and Mitochondrial Function Are Correlated With Frailty in Elderly Men

**DOI:** 10.3389/fmed.2022.816045

**Published:** 2022-01-28

**Authors:** Li Meng, Hong Shi, Da-guang Wang, Jing Shi, Wen-bin Wu, Ya-min Dang, Guo-qing Fan, Ji Shen, Pu-lin Yu, Jun Dong, Rui-yue Yang, Huan Xi

**Affiliations:** ^1^The Key Laboratory of Geriatrics, Beijing Institute of Geriatrics, Institute of Geriatric Medicine, Beijing Hospital/National Center of Gerontology of National Health Commission, Chinese Academy of Medical Sciences, Beijing, China; ^2^Department of Geriatrics, National Clinical Research Center for Geriatrics, National Center of Gerontology, Institute of Geriatric Medicine, Beijing Hospital, Chinese Academy of Medical Sciences, Beijing, China; ^3^Department of Laboratory Medicine, National Center of Gerontology, Institute of Geriatric Medicine, Beijing Hospital, Chinese Academy of Medical Sciences, Beijing, China

**Keywords:** frailty, sarcopenia, metabolomics, multivariate analysis, biomarker

## Abstract

**Background:**

As an age-related syndrome, frailty may play a central role in poor health among older adults. Sarcopenia overlaps with the physical domain of frailty, and most existing studies have analyzed the associated factors of frailty and sarcopenia as an isolated state. Perturbations in metabolism may play an important role in the presence of frailty or sarcopenia; however, the metabolites associated with frailty, especially overlapping with sarcopenia remain unclear. In this study, we aimed to explore whether amino acids, carnitines, acylcarnitines and lysophosphatidylcholines, as specific panels, are significantly correlated with frailty, especially overlapping with sarcopenia, to gain insight into potential biomarkers and possible biological mechanisms and to facilitate their management.

**Methods:**

We applied a targeted high-performance liquid chromatography-tandem mass spectrometry approach in serum samples from 246 Chinese older men (age 79.2 ± 7.8 years) with frailty (*n* = 150), non-frailty (*n* = 96), frailty and sarcopenia (*n* = 52), non-frail and non-sarcopenic control (*n* = 85). Frailty was evaluated using Freid phenotype criteria, sarcopenia was defined by diagnostic algorithm of Asian Working Group on Sarcopenia, and the participants were diagnosed as frailty and sarcopenia when they met the evaluation criteria of both frailty and sarcopenia. A panel of 29 metabolomic profiles was assayed and included different classes of amino acids, carnitines, acylcarnitines, and lysophosphatidylcholines (LPCs). Multivariate logistic regression was used to screen the metabolic factors contributing to frailty status, and orthogonal partial least squares discriminant analysis was used to explore important factors and distinguish different groups.

**Results:**

In older men demonstrating the frail phenotype, amino acid perturbations included lower tryptophan and higher glycine levels. With regard to lipid metabolism, the frailty phenotype was characterized by lower concentrations of isovalerylcarnitine (C5), LPC16:0 and LPC18:2, while higher levels of octanoyl-L-carnitine (C8), decanoyl-L-carnitine (C10), dodecanoyl-L-carnitine (C12) and tetradecanoyl-L-carnitine (C14). After adjusting for several clinical confounders, tryptophan, LPC18:2, LPC 16:0 and C5 were negatively correlated with frailty, and C8 and C12 were positively related to frailty. We preliminarily identified metabolic profiles (LPC16:0, LPC18:2, glycine and tryptophan) that may distinguish older men with frailty from those without frailty. Importantly, a set of serum amino acids and LPCs (LPC16:0, LPC18:2, and tryptophan) was characterized in the metabotype of older adults with an overlap of frailty and sarcopenia. The metabolites that were most discriminating of frailty status implied that the underlying mechanism might be involved in antioxidation and mitochondrial dysfunction.

**Conclusions:**

These present metabolic analyses may provide valuable information on the potential biomarkers and possible biological mechanisms of frailty, and overlapping sarcopenia. The findings obtained may offer insight into their management in older adults.

## Introduction

As an age-related clinical syndrome, frailty is caused by a progressive discrepancy between the accumulation of damage and resilience of the body, and can explain some of the health heterogeneity among people with the same chronological age (1). Frailty-related adverse consequences in older adults such as falls, dependency, hospitalization, and death result in increased medical costs and demand for long-term care (2). With respect to rapidly aging populations in China, the overall weighted prevalence of frailty is 9.9%, and increases with age (3, 4). Moreover, sarcopenia overlaps with the physical domain of frailty and may represent the biological substratum of complex pathophysiology. In patients aged 70 years or older, the concurrent occurrence of frailty and sarcopenia syndromes (overlap) is 19% (5). Other studies have indicated that 58% of frail elderly individuals have sarcopenia, and men have higher prevalence of sarcopenia than women in China (6, 7), which may challenge their management.

The biological mechanisms underlying frailty have been extensively studied in recent years. Scientific progress in understanding frailty has been hampered by disagreement about its pathophysiology and operational diagnosis in research and clinical settings (8). Frailty encompasses several multisystem derangements, which means that there is no single diagnostic tool or biomarker available to identify the presence and extent of frailty. Advances in metabolism and other omics platforms have provided new information on the molecular mechanisms underlying frailty. Circulating metabolites have emerged as a possible tool for capturing this complexity and hold the potential to identify metabolic factors or biomarkers that contribute to frailty, offering opportunities to develop strategies for its identification and management (9, 10). However, there is a lack of stable and reliable frailty-related biomarkers for use in clinical practice (11, 12). Moreover, the various biomarkers for frailty thus far discovered in metabolomics have focused mostly on amino acids (13, 14), and relatively less is known about the association of frailty with lipid metabilism such as carnitine, acylcarnitines (AcyCNs) and lysophosphatidylcholines (LPCs), in part because of their structural diversity and the sheer number of discrete molecular species (15). Carnitines and AcyCNs may play some role in muscle weakness, cognitive diseases, and inflammatory conditions (16, 17), whereas, the circulating LPCs content has been used to predict a decline in gait speed or cognitive impairment (18–20). It is therefore important to carry out extensive measurements of these metabolic species to further our insights into the metabolic basis of frailty. However, the metabolites associated with sarcopenia have not been comprehensively profiled and examined. Most of existing studies have analyzed the associated factors of frailty and sarcopenia as isolated states, and only a few studies have merged the two conditions into one entity (i.e., overlapped frailty and sarcopenia; F&S) (21). Although some studies on frailty or sarcopenia with investigation of metabolism have been conducted in other countries, limited data are available about the association of metabolites with frailty and sarcopenia in older Chinese adults.

With the aim of expanding knowledge in this area, in the current study, we determined the serum metabolomic profile using targeted tandem mass spectrometry coupled with multivariate statistical analysis, to simultaneously analyze different classes of amino acids, LPCs, carnitines and AcyCNs in older Chinese men. We explored the metabolic factors of frailty, and then narrowed down the range to study the discriminating metabolites of frailty overlapped with sarcopenia. We aimed to explore whether amino acids, carnitine, AcyCNs and LPCs, as specific panels, are significantly correlated with frailty, especially F&S, to gain insight into possible biological mechanisms, as well as to facilitate the management of frailty.

## Materials and Methods

### Participants

A total of 246 elderly men (62–100 years old) attending the Geriatric Medicine department of Beijing Hospital for physical examination from August 2016 to May 2018 were recruited using random numbers after the inclusion and exclusion. The inclusion criteria were as follows: participants were older than age 60 years, had the ability to understand and complete the questionnaires, and did not have severe mental or cognitive disorders. People with malignant tumors, blood system diseases, chronic obstructive pulmonary disease, autoimmune diseases, infectious diseases, and individuals who were taking amino acid supplements were excluded. This study was approved by the Medical Ethics Committee of Beijing Hospital of the National Health Commission.

In order to assess the health status related to frailty or sarcopenia, we administered comprehensive geriatric assessment consisting of general clinical information and demographic data. Each participant self-reported their smoking and alcohol drinking status (past, present or never smoker/alcohol intake). Polypharmacy was defined as the use of more than five medications (22). Comorbidities were slightly modified from the Charlson Comorbidity Score (23), and the following comorbidities were retained: hypertension, coronary artery disease, arrhythmia, diabetes mellitus, peripheral vascular disease, cerebrovascular disease, stroke, chronic pulmonary disease, kidney disease, prostatic disease, gastric ulcer, liver disease and osteoporosis. The presence of depressive symptoms was defined using the Geriatric Depression Scale-5; cognitive function was evaluated with Mini–Mental State Examination and malnutrition risk was evaluated using the Mini-Nutritional Assessment Short-Form. Participants were assessed for dependence in six basic activities of daily living (ADL) and eight instrumental ADL tasks.

### Frailty and Sarcopenia Assessment

In this study, frailty was defined according to the Fried phenotype criteria, which include exhaustion, weakness, slowness, physical inactivity and weight loss (24). Exhaustion was defined using the two statements of the Center for Epidemiologic Studies-Depression scale. The maximum handgrip strength of either hand was measured two times using a digital dynamometer (WCS-II, Beijing) for assessment of weakness. Slowness was evaluated using the average of two timed- walk tests over a 6 m course. Physical activity was evaluated using the Chinese version of the International Physical Activity Questionnaire (25). Weight loss was defined as having involuntary weight loss of >5% in the previous year or 3 kg within the past 3 months. Older adults were considered to be frail if they met three or more of these five criteria, prefrail if they met one or two criteria, and non-frailty if they met none of these criteria. A combined group of prefrail together with frail was analyzed as the “frailty” group (non-frailty was the reference group) because of the small sample size of the frail group, according to the previous study.

We defined sarcopenia using the diagnostic algorithm of the Asian Working Group on Sarcopenia (AWGS) according to the presence of both low muscle mass and low muscle function (slow walking speed or low grip strength) (26). Appendicular skeletal muscle mass (ASM) was determined using a bioelectrical impedance data acquisition system (Inbody 720; BiospaceCo, Ltd, Seoul, Korea). The skeletal muscle mass index (SMI) or relative ASM was calculated as ASM divided by height (m) squared (ASM/height^2^, kg/m^2^). Low muscle strength was defined as handgrip strength < 28 kg for men and low physical performance was defined as a 6-m walk speed < 1.0 m/s. The cutoff for height-adjusted muscle mass (i.e., SMI) *via* bioelectrical impedance data was <7.0 kg/m^2^ in men. Participants were diagnosed with F&S when they met the evaluation criteria of both frailty and sarcopenia; non-frail and non-sarcopenic control did not meet the criteria for either sarcopenia or frailty (27).

### Sample Preparation and Routine Chemical Parameters

Blood samples were collected in the morning using venipuncture of the median cubital vein after participants had completed an overnight fast. For serum separation, samples were left at room temperature for 20 min and subsequently centrifuged at 1,000 × *g* for 10 min at 4°C. Aliquots of serum were subsequently stored at –80°C until analysis. Blood samples were measured using an automatic biochemical analyzer and enzymatic colorimetric tests for laboratory routine parameters.

### Measurements of Serum Metabolites

A panel of 29 Serum metabolites was measured using our previously reported targeted liquid chromatography-tandem mass spectrometry method for simultaneous quantification of three branched-chain amino acids (BCAAs; leucine, isoleucine, valine), three aromatic amino acids (AAAs; phenylalanine, tryptophan, and tyrosine), glutamate (Glu), glutamine (Gln), Gln/Glu ratio, alanine, glycine, four subclasses of LPC, carnitine, and 12 species of AcyCN, trimethylamine-N-oxide (TMAO) and betaine (28, 29). Briefly, 0.01 mL aliquots of the calibrators or serum samples were mixed with 0.01 mL of isotopically labeled internal standards, followed by the addition of 1 mL of isopropanol. After vortexing and centrifuging, 0.2 mL of the supernatant was transferred to another vial and reconstituted. LC–MS/MS analysis was performed using an AB Sciex 5500 QTRAP tandem mass spectrometer (Framingham, MA, USA) equipped with an Agilent 1260 Series high-performance liquid chromatography system (Santa Clara, CA, USA).

### Statistical Analyses

Continuous variables are expressed as mean ± standard deviation or median (25th percentile, 75th percentile; P25, P75), and categorical data are expressed as percentage. The normal distribution of data was ascertained using the Kolmogorov–Smirnov test. Metabolites with a skewed distribution were log-transformed. Comparisons between two groups for normally distributed continuous variables were performed using the *t*-test. The non-parametric Wilcoxon test was applied to assess differences in non-normally distributed continuous data. Differences in categorical variables between groups were determined *via* χ^2^ or Fisher's exact tests. A Benjamini–Hochberg correction was used to account for multiple comparisons. Significance levels in prior exploratory analyses (30, 31) guided the choice of statistical significance in this study, which was set at *p* < 0.05 with a false discovery rates (FDR) ≤ 0.30.

Spearman correlations were used to examine the relationship between metabolites. In multivariable logistic models of metabolites and frailty, clinical potential confounding covariates, that were significantly associated with frailty in the above univariate analyses and previous studies (32–34) were adjusted in three models, as follows: Model 1: age, body mass index, and smoking status; Model 2: further controlled for malnutrition, cognitive decline, ADL, polypharmacy, and comorbidities; Model3: further controlled for red blood cell count and hemoglobin, alanine aminotransferase (ALT), and uric acid. Statistical analyses were performed using the SAS statistical package, version 9.3 (SAS institute Inc., Cary, NC, USA). All tests were two-sided, with statistical significance set at *p* < 0.05.

Orthogonal partial least squares discriminant analysis (OPLS-DA) in SIMCA-P v.14.1 was applied to distinguish those metabolites related to frailty and to enhance the separation between groups using rotating principal component analysis. Among the possible classifiers, OPLS-DA was selected because of its versatility and ability to deal with highly correlated predictors (35–37) and because it is more effective in focusing the correlated information onto the first predictive component instead of scattering it onto the subsequent components. The quality of the OPLS-DA model was validated using cross-validation: the goodness of fit (R2) and the goodness of prediction indicated by the cumulated Q2 value (Q2 cum). Permutation tests were used to assess whether the model was overfitted. The area under the receiver operating characteristic (ROC) curve can be used as an indicator of discriminatory power of the model. The important factors were identified using S-Plot and variable importance in projection (VIP) score. A VIP score > 1 indicated that the independent variable was an important factor in distinguishing frailty status. Metabolites with VIP > 1 were further subjected to univariate analysis to measure the significance of each metabolite.

## Results

### Clinical Characteristics of the Study Participants

The participants included in the analysis comprised 246 men aged 62–100 years. According to the Fried phenotype criteria, the participants were stratified into 96 non-frailty (39.0%), 132 prefrail men (53.7%) and 18 frail men (7.3%). In this study, prefrail and frail men were combined into the frailty group (150, 61.0%). In terms of frailty criteria constituents, 57 men (51.8%) in the frail group and 15 men (15.96%) in the non-frailty group had lower grip strength. The frailty group had a slower walking speed, greater reductions in physical activity, and a higher proportion of participants with exhaustion than the non-frailty group. Similarly, the SMI was smaller in the frailty group than in the non-frailty group. Study participants included 65 (26.4%) older men with sarcopenia and 181 (73.6%) non-sarcopenic elderly men; moreover, 52 (21.1%) men had F&S, whereas, 85 (34.6%) men were non-frailty and non-sarcopenia (non-frail and non-sarcopenic control).

The main clinical and functional characteristics of the study population are presented in Table 1. The frailty group was older than non-frailty group. Compared with non-frailty participants, frailty participants had a lower body mass index (BMI), were less likely to smoke, and had lower diastolic blood pressure. No participants were classified as ADL-dependent in non-frailty group, whereas 21.6% of frailty men were ADL-dependent. Men in the frail group more frequently had cognitive decline, comorbidities and malnutrition in comparison with non-frailty men. However, polypharmarcy and depression did not differ significantly between the two groups.

**Table 1:** Clinical characteristics of elderly individuals with frail and non-frail status.

**Variables**	**Non-frailty (*n* = 96)**	**Frailty (*n* = 150)**	**t/χ^2^/Z**	***p*-value**
Age (years)^a^	78.01 ± 6.99	79.94 ± 8.21	−2.04	**0.042**
past/present smoking	25.0%	12.7%	6.173	**0.013**
Alcohol intake	20.62%	13.64%	2.123	0.145
BMI (kg/m^2^)^b^	26 (23, 27)	25 (22, 27)	2.351	**0.019**
Diastolic BP (mmHg)^b^	75 (70, 80)	70 (65, 80)	3.044	**0.002**
Systolic BP (mmHg)^b^	130 (120, 140)	130 (120, 140)	0.082	0.934
Malnutrition	18.63%	33.95%	7.285	**0.007**
Comorbidities^a^	3.5 ± 1.4	4.2 ± 1.8	3.235	**0.001**
Polypharmacy	82.61%	85.33%	Fisher	0.747
ADL decline	0.00%	21.60%	25.405	**<0.001**
Cognitive decline	0.98%	7.41%	5.522	**0.019**
Depression	41.18%	50.62%	2.24	0.135
Slowness	22.77%	57.24%	29.329	**<0.001**
Weakness	15.96%	51.82%	28.542	**<0.001**
Lower SMI	21.43%	39.53%	8.43	**0.004**
Lower activity	4.95%	29.11%	22.749	**<0.001**
Exhaustion	0.01%	12.96%	14.365	**<0.001**
**Blood general parameters**
Red blood cell (10^12^/L)^b^	4.69 (4.49, 4.93)	4.55 (4.22, 4.94)	2.469	**0.014**
White blood cell (10^9^/L)^a^	5.89 ± 1.21	6.03 ± 1.71	−0.26	0.795^c^
Platelets (10^9^/L)^b^	187 (162, 215)	183 (159, 211)	0.862	0.389
Hemiglobin (g/L)^b^	144.3 (138.0, 151.0)	139.0 (131.0, 148)	3.289	**0.001**
Fasting glucose (mmol/L)^b^	5.8 (5.3, 6.5)	5.7 (5.3, 6.2)	0.516	0.606
HbALc (%)^b^	5.85 (5.50, 5.92)	5.8 (5.60, 6.37)	−1.772	0.076
Triglyceride (mmol/L)	1.3 (0.92, 1.75)	1.21 (0.83, 1.69)	1.103	0.270
Total cholesterol (mmol/L)^b^	4.22 (3.38, 5.00)	4.13 (3.33, 4.84)	0.738	0.461
LDL-C (mmol/L)^b^	2.45 (1.83, 3.08)	2.34 (1.81, 2.86)	0.983	0.325
HDL-C (mmol/L)^b^	1.19 (0.99, 1.35)	1.22 (1.03, 1.37)	−0.504	0.614
ALT (U/L)^b^	19 (16, 24)	17 (14, 22)	1.964	**0.049**
AST (U/L)^b^	27 (23, 30)	27 (24, 31)	−0.417	0.677
Albumin (g/L)^a^	42.53 ± 1.58	42.13 ± 1.82	1.748	0.080
Uric acid (μmo/L)^b^	369.6 (312, 443)	342 (298, 397)	1.983	**0.047**
Creatinine (μmo/L)^b^	77 (68, 88)	77 (68, 93)	−0.870	0.384
BUN (mmo/L)^a^	5.57 ± 1.21	6.05 ± 1.86	−1.92	0.056^c^

a *Mean ± standard deviation*.

b *Median and interquartile range (P25, P75)*.

c *After log transformation*.

*Bold values mean statistical significance (p < 0.05). ADL, activities of daily living; BMI, body mass index; HbALc, glycosylated hemoglobin; LDL-C, low density lipoprotein cholesterol; HDL-C, high density lipoprotein cholesterol; ALT, glutamate pyruvate transaminase; AST, aspartate aminotransferase; BUN, blood urea nitrogen; SMI, skeletal muscle index*.

With respect to routine chemistry parameters, frailty participants had lower concentrations of red blood cells and hemoglobin. Serum uric acid and ALT levels in frailty participants were significantly lower than those in non-frailty participants. No other blood chemical parameters, such as albumin, fasting blood glucose, triglycerides, total cholesterol, low-density lipoprotein cholesterol, high-density lipoprotein cholesterol, and aspartate aminotransferase were found to be significantly different between the two groups.

### Differences in Serum Concentration of Metabolites Between With and Without Frailty

A description of metabolic species levels is provided in Table 2. The frailty phenotype showed specific amino acid alterations. The serum concentration of tryptophan was significantly lower in the frailty group; in contrast, the concentration of glycine was markedly higher relative to that in the non-frailty group. We detected no significant difference in other amino acids, such as BCAAs, Glu, Gln, and other AAAs. Among AcyCNs in the frailty group, the concentrations of octanoyl-L-carnitine (C8), decanoyl-L-carnitine (C10), dodecanoyl-L-carnitine (C12), and tetradecanoyl-L-carnitine (C14) were higher, whereas that of isovalerylcarnitine (C5) was lower compared with those observed in the non-frailty group; however, these did not remain significant after multiple adjustments. This change trend was not found in other AcyCN species. The perturbation of serum LPC occurring in frailty individuals was evident, especially for lower levels of 1-palmitoyl-2-hydroxy-sn-glycero-3-phosphocholine (LPC16:0) and 1-linoleoyl-2-hydroxy-sn-glycero-3-phosphocholine (LPC18:2) subclasses. Serum levels of trimethylamine-N-oxide and betaine showed no significant differences between the two groups.

**Table 2:** Serum concentrations of metabolites in participants with and without frailty.

**Variables**	**Non-frailty (*n* = 96)**	**Frailty (*n* = 150)**	**t/Z**	***p*-value**	**FDR**
Valine^a^	39.9 ± 5.9	39.0 ± 6.8	1.09	0.276	0.037
Ileucine^a^	12.7 ± 2.4	12.4 ± 2.4	0.93	0.354^c^	0.305
Leucine^a^	25.9 ± 3.9	25.3 ± 4.4	1.29	0.198^c^	0.194
Tyrosine^a^	16.2 ± 2.7	15.5 ± 3.3	1.96	0.051	0.245
Phenylalanine^a^	19.4 ± 3.3	19.6 ± 4.0	−0.07	0.942^c^	0.740
Tryptophan^a^	17.6 ± 2.5	16.6 ± 3.2	3.25	**0.001** ^c^	**0.185**
Glutamine^a^	26.1 ± 7.3	26.3 ± 6.9	−0.30	0.761^c^	0.480
Glutamate^a^	22.2 ± 3.8	21.8 ± 4.9	1.25	0.212^c^	0.335
Glycine^b^	37.38 (32.49, 43.45)	40.07 (35.36, 48.19)	−2.393	**0.017**	**0.048**
Alanine^a^	50.3 ± 10.3	49.8 ± 10.9	0.46	0.649^c^	0.309
Gln/Glu	1.2 ± 0.4	1.3 ± 0.5	−0.61	0.545^c^	0.392
BCAA^a^	78.7 ± 11.8	76.8 ± 12.8	1.25	0.213	0.336
AAA^a^	53.0 ± 6.6	51.8 ± 9.1	1.20	0.230	0.254
Carnitine (μg/mg)^a^	10.6 ± 1.9	10.2 ± 2.2	1.74	0.084^c^	0.287
**Acylcarnitines (μg/mg)**
C2^a^	2.2 ± 0.7	2.2 ± 0.7	0.30	0.766^c^	0.378
C3^a^	0.1 ± 0.0	0.1 ± 0.0	1.02	0.308^c^	0.715
C5^b^	0.028 (0.020, 0.034)	0.024 (0.018, 0.029)	3.322	**0.001**	0.441
C6^b^	0.021 (0.016, 0.025)	0.021 (0.017, 0.027)	−0.884	0.377	0.727
C8^b^	0.057 (0.042, 0.075)	0.066 (0.050, 0.082)	−2.513	**0.012**	0.808
C10^b^	0.088 (0.061, 0.115)	0.095 (0.075, 0.128)	−2.016	**0.044**	0.763
C10:1^b^	0.118 (0.10, 0.160)	0.132 (0.102, 0.181)	−1.695	0.090	0.745
C12^b^	0.027 (0.020, 0.035)	0.029 (0.024, 0.038)	−2.06	**0.040** ^c^	0.833
C14^b^	0.01 (0.008, 0.013)	0.011 (0.009, 0.014)	−2.30	**0.022** ^c^	0.785
C16^b^	0.054 (0.048, 0.061)	0.053 (0.046, 0.062)	0.13	0.896^c^	0.744
C18^a^	0.0245 (0.022, 0.028)	0.024 (0.02, 0.028)	1.35	0.177^c^	0.753
C18:1^b^	0.098 (0.084, 0.113)	0.0925 (0.082, 0.109)	1.528	0126	0.772
LPC16:0 (μg/ml)^b^	121.07 (95.71, 148.01)	105.21 (84.55, 129.62)	2.929	**0.003**	**0.205**
LPC18:0 (μg/ml)^b^	37.09 (31.30, 41.97)	36.55 (30.73, 42.48)	−0094	0.925	0.247
LPC18:1 (μg/ml)^a^	17.9 ± 3.7	17.3 ± 4.2	1.48	0.140	0.221
LPC18:2 (μg/ml)^b^	30.11 (22.54, 36.06)	24.14 (17.46, 33.40)	3.296	**0.001**	**0.022**
TMAO (μg/ml)^b^	0.40 (0.26, 0.56)	0.46 (0.28, 0.73)	−1.915	0.056	0.478
Betaine (μg/ml)^b^	7.65 (6.37, 8.66)	7.886 (6.41, 9.35)	−0.070	0.481	0.458

a *Mean ± standard deviation*.

b *Median and interquartile range (P25, P75)*.

c *After log transformation*.

*Bold values mean statistical significance (p < 0.05), and false discovery rates (FDR) ≤ 0.30. BCAA, branched-chain-aminoacids; AAA, aromatic-aminoacids; C2, acetylcarnitine; C3, propionyl-L-carnitine; C5, valeryl-L-carnitine; C6, AcyCNhexenoyl-L-carnitine; C8, octanoyl-L-carnitine; C10, decanoyl-L-carnitin; C10:1, decenoyl-L-carnitine; C12, dodecanoy-L-carnitine; C14, tetradecanoyl-L-car-nitine; C16, hexadec-anoyl-L-carnitine; C18, octadecanoyl-L-carnitine; C18:1, octadecenoyl-L-carnitine; LPC16:0, 1-palmitoyl-2-hydroxy-sn-glycero-3-phosphocholine; LPC18:0, 1-stearoyl-2-hydroxy-sn-glycero-3-phosphocholine; LPC18:1, 1-oleoyl-2-hydroxy-sn-glycero-3-phosphocholine; LPC18:2, 1-linoleoyl-2-hydroxy-sn-glycero-3-phosphocholine; TMAO, trimethylamine oxid*.

### Correlation Analyses Between the Significant Metabolites

**Figure 1:**
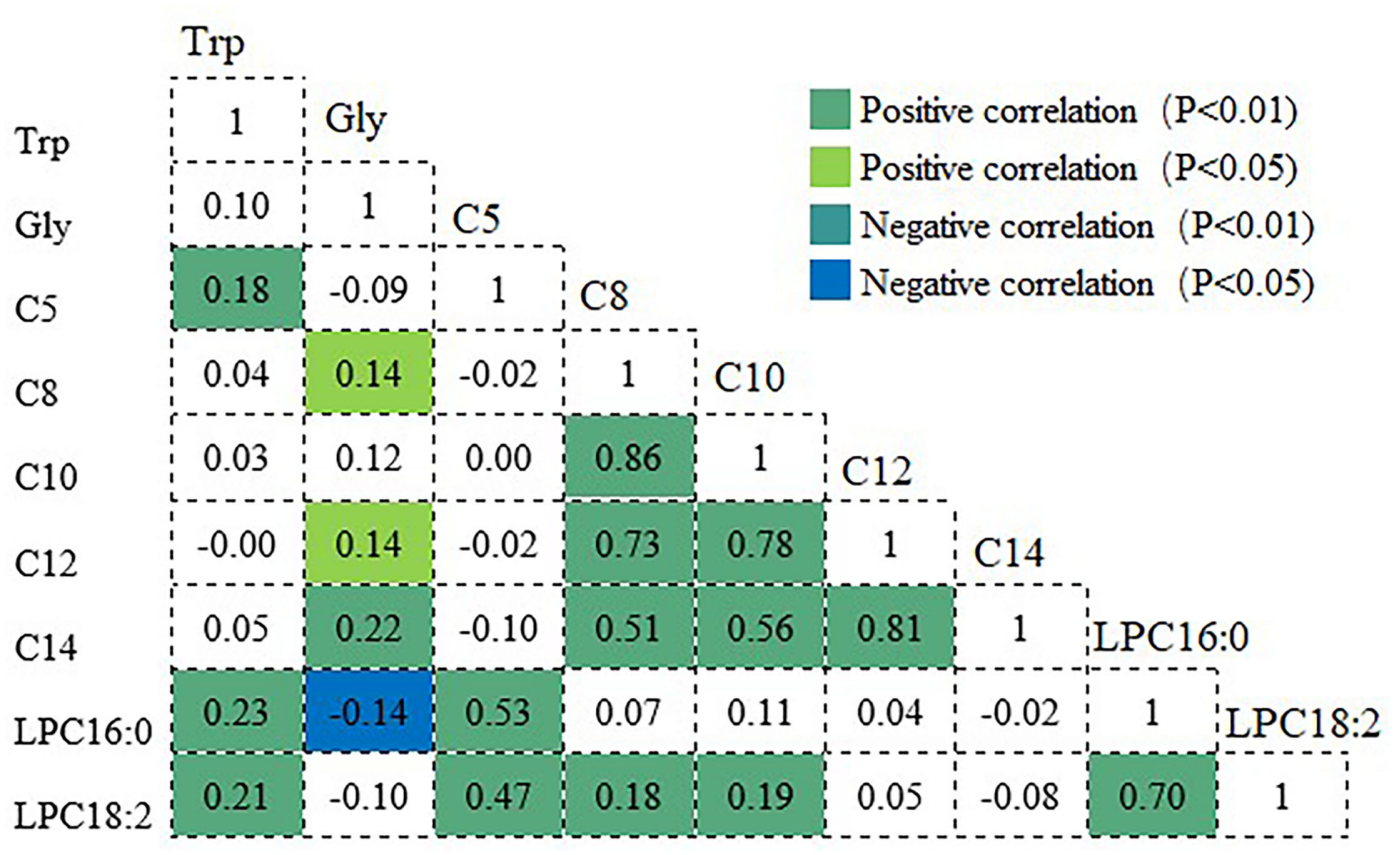
Correlation coefficients among the metabolites significantly associated with frailty. Positive and negative correlations are shown in green and blue, respectively. Trp, tryptophan; Gly, glycine; C5, valeryl-L-carnitine; C8, octanoyl-L-carnitine; C10, decanoyl-L-carnitin; C12, dodecanoy-L-carnitine; C14, tetradecanoyl-L-car-nitine; LPC16:0, 1-palmitoyl-2-hydroxy-sn-glycero-3-phosphocholine; LPC18:2, 1-linoleoyl-2-hydroxy-sn-glycero-3-phosphocholine.

Figure 1 shows the correlations among metabolites that were significantly relevant to frailty. Variations in these metabolites were not independent, and many showed significant intercorrelations with one another. Tryptophan showed positive associations with C5 and slight associations with LPC16:0 and LPC18:2. In turn, C5 was moderately associated with LPC16:0 and LPC18:2. Glycine presented significant positive associations with C8, C12, and C14 and inverse associations with LPC16:0, whereas, a significant positive correlation between LPC16:0 and LPC18:2 was found (*r* = 0.70).

Significant correlations were also observed between acylcarnitines, indicating that they change in ways that are interrelated. Very strong positive correlations between C8 and C10, and between C12 and C14 were observed, and moderate correlations were observed between C8 and C14 and between C10 and C14. The correlation coefficients of C8 with C10 and C12 were *r* = 0.86 and 0.73, respectively, whereas, those of C10 with C12, and of C12 with C14 were r = 0.78 and 0.81, respectively.

### Metabolic Factors Associated With Frailty Status in Logistic Regression Analysis

Table 3 summarizes the results of logistic regression analyses. Multivariate adjustment was used to control for clinical factors, including age, BMI, current smoking, malnutrition, cognitive decline, ADL, polypharmacy, comorbidities, red blood cell count, hemoglobin, ALT and uric acid. After adjusting for these clinical factors, the levels of tryptophan, C8, C12, LPC16:0, LPC18:2, and C5 were found to be independent factors for frailty. When these metabolites were used as categorical variables, the odds ratio (OR) (95% CI) of the second quartile was 0.278 (0.118–0.655) for tryptophan, that of the second quartile for C8 was 2.435 (1.066–5.564), that of the lower quartile for C12 was 4.059 (1.919–8.586), that of the third quartile for LPC16:0 was 0.322 (0.136–0.76), and that of the second quartile for LPC18:2 was 0.18 (0.072–0.447), compared with the lowest quartile. Additionally, when analyzed as continuous variables, C5, LPC16:0, and LPC18:2 were dependently associated with frailty in three adjustment models (all *p* < 0.05 Supplementary Table 1).

**Table 3:** Logistic regression analysis for significant associations of serum metabolites with frailty.

**Variables**	**Model1**				**Model 2**				**Model 3**		
	*****β*****	***SE***	***OR* (95% *CI*)**	***P***	**β**	***SE***	***OR* (95% *CI*)**	***P***	*****β*****	***SE***	***OR* (95% *CI*)**	***P***
Tryptophan -M3	−0.612	0.395	0.542 (0.250, 1.177)	0.122	−0.325	0.432	0.722 (0.310, 1.684)	0.451	−0.298	0.446	0.743 (0.310, 1.780)	0.505
-M2	−1.378	0.391	0.252 (0.117, 0.543)	**0.000**	−1.270	0.428	0.281 (0.121, 0.65)	**0.003**	−1.282	0.438	0.278 (0.118, 0.655)	**0.003**
-M1	−0.312	0.401	0.732 (0.334, 1.607)	0.437	−0.382	0.441	0.683 (0.288, 1.62)	0.387	−0.297	0.453	0.743 (0.306, 1.805)	0.512
C8-M3	0.909	0.394	2.481 (1.147, 5.367)	**0.021**	0.788	0.432	2.198 (0.942, 5.129)	0.069	0.789	0.454	2.202 (0.905, 5.359)	0.082
-M2	0.864	0.376	2.373 (1.135, 4.962)	**0.022**	0.809	0.411	2.245 (1.002, 5.027)	**0.049**	0.890	0.422	2.435 (1.066, 5.564)	**0.035**
-M1	0.507	0.366	1.661 (0.811, 3.402)	0.166	0.382	0.400	1.465 (0.669, 3.206)	0.340	0.248	0.413	1.282 (0.571, 2.879)	0.548
C12-M3	0.201	0.439	1.222 (0.517, 2.887)	0.647	0.322	0.486	1.38 (0.533, 3.577)	0.507	0.267	0.495	1.305 (0.495, 3.443)	0.590
-M2	0.430	0.445	1.538 (0.643, 3.679)	0.333	0.244	0.492	1.276 (0.486, 3.349)	0.621	0.301	0.523	1.351 (0.485, 3.762)	0.565
-M1	1.212	0.335	3.36 (1.741, 6.484)	**0.000**	1.249	0.369	3.488 (1.692, 7.191)	**0.001**	1.401	0.382	4.059 (1.919, 8.586)	**0.000**
LPC16:0-M3	−0.852	0.374	0.427 (0.205, 0.887)	**0.023**	−0.850	0.413	0.427 (0.190, 0.960)	**0.040**	−1.134	0.438	0.322 (0.136, 0.76)	**0.010**
-M2	−0.323	0.378	0.724 (0.345, 1.518)	0.393	−0.193	0.412	0.824 (0.368, 1.848)	0.639	−0.294	0.433	0.745 (0.319, 1.74)	0.496
-M1	0.084	0.392	1.087 (0.505, 2.344)	0.831	0.109	0.429	1.116 (0.481, 2.588)	0.799	0.014	0.443	1.014 (0.426, 2.418)	0.974
LPC18:2-M3	−0.715	0.389	0.489 (0.228, 1.048)	0.066	−0.749	0.427	0.473 (0.205, 1.092)	0.079	−1.171	0.460	0.31 (0.126, 0.763)	**0.011**
-M2	−1.225	0.386	0.294 (0.138, 0.625)	**0.002**	−1.362	0.434	0.256 (0.109, 0.600)	**0.002**	−1.718	0.466	0.18 (0.072, 0.447)	**0.000**
-M1	−0.080	0.401	0.923 (0.421, 2.025)	0.841	−0.078	0.439	0.925 (0.392, 2.184)	0.858	−0.336	0.462	0.714 (0.289, 1.765)	0.466

*Model1: age, body mass index, smoking status; Model2: further controlled for malnutrition, cognitive decline, activities of daily living, polypharmacy, comorbidities; Model3: further controlled for red blood cell and hemoglobin, alanine aminotransferase and uric acid; Measured metabolites were used as categorical variables: lower quartile (M1), median (M2), upper quartile (M3) vs. lowest quartile. C5, valeryl-L-carnitine; C8, octanoyl-L-carnitine; C10, decanoyl-L-carnitin; C12, dodecanoy-L-carnitine; C14, tetradecanoyl-L-car-nitine; LPC16:0, 1-palmitoyl-2-hydroxy-sn-glycero-3-phosphocholine; LPC18:2, 1-linoleoyl-2-hydroxy-sn-glycero-3-phosphocholine. Bold values mean statistical significance (p < 0.05)*.

No significant relationship was found between levels of glycine, C10, C14, and frailty in Models 2 and 3, whereas, a significant association was observed in Model 1 (Supplementary Table 2). When the data were examined, taking into account malnutrition and chemical indices, the results for tryptophan, C8, and LPC16:0 were changed slightly and remained significantly associated with frailty; however, the effects of glycine, C10, and C14 on the presence of frailty were reduced or remained non-significant after further adjustment.

### Orthogonal Partial Least Squares Discriminant Analysis of Frailty and Sarcopenia

**Figure 2:**
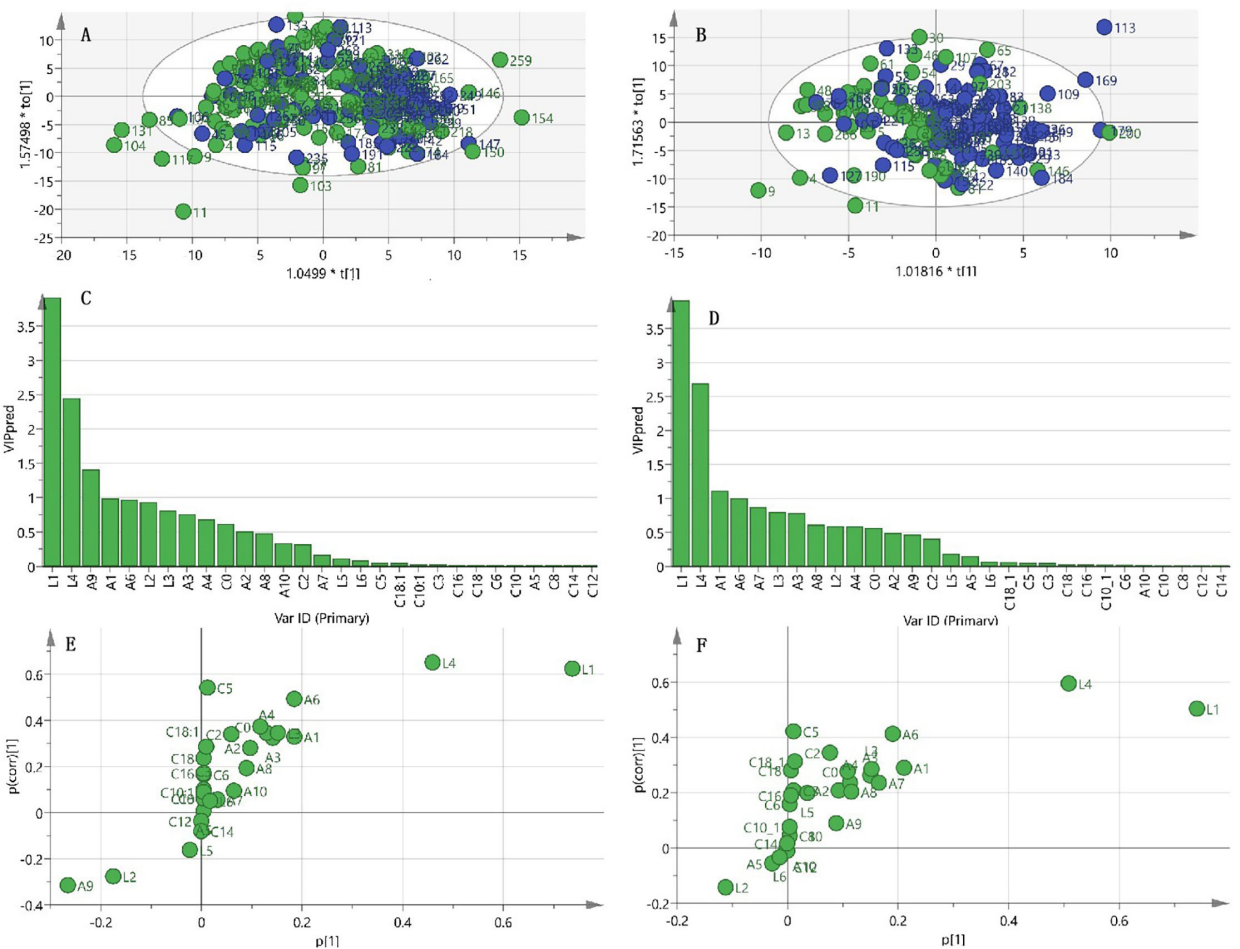
OPLS-DA analysis for frailty status using metabolism data. **(A)** Scatter plot of OPLS-DA model analysis for frailty (green circle) vs. non-frailty groups (blue circle) by the main components. **(B)** Scatter plot of OPLS-DA model analysis for frailty and sarcopenia (F&S) (green circle) vs. non-frail and non-sarcopenic control (blue circle) by the main components. **(C)** Ordered list of metabolites with higher discrimination ability based on variable importance for the projection (VIP) score for separation of frailty and non-frailty groups. **(D)** Ordered list of metabolites with higher discrimination ability based on VIP for separation of frailty and sarcopenia (F&S) vs. non-frail and non-sarcopenic control. **(E)** S-plot of the OPLS-DA model of the frailty and non-frailty groups. **(F)** S-plot of the OPLS-DA model of frailty and sarcopenia vs. the non-frail and non-sarcopenic control. A9, glycine; A1, valine; A6, tryptophan; A3, leucine; A4, tyrosine; A2, Ileucine; A8, glutamate; A10, alanine; A7, glutamine; A5, phenylalanine; C0, carnitine; C2, acetylcarnitine; C3, propionyl-L-carnitine; C5, valeryl-L-carnitine; C6, AcyCNhexenoyl-L-carnitine; C8, octanoyl-L-carnitine; C10, decanoyl-L-carnitin; C10:1, decenoyl-L-carnitine; C12, dodecanoy-L-carnitine; C14, tetradecanoyl-L-car-nitine; C16, hexadec-C16anoyl-L-carnitine; C18, octadecanoyl-L-carnitine; C18:1, octadecenoyl-L-carnitine; L1, 1-palmitoyl-2-hydroxy-sn-glycero-3-phosphocholine (LPC16:0); L2, 1-stearoyl-2-hydroxy-sn-glycero-3-phosphocholine (LPC18:0); L3, 1-oleoyl-2-hydroxy-sn-glycero-3-phosphocholine (LPC18:1); L4, 1-linoleoyl-2-hydroxy-sn-glycero-3-phosphocholine (LPC18:2); L5, trimethylamine-N-oxide (TMAO); L6, betaine.

To better understand the correlation between measured metabolic factors and frailty status, OPLS-DA was performed to identify the main components that could discriminate the two groups. Unfortunately, the OPLS-DA score plot yielded no clear separation and merely showed a trend distinguishing frailty elderly men from their non-frailty counterparts (Figure 2A). The parameters of goodness of fit and goodness of prediction in the OPLS-DA model showed that this model had a certain ability to explain but low ability to predict frailty (R2X = 0.517, R2Y = 0.231, Q2 = 0.025), suggesting that it was not optimal for discriminating the two groups with these metabolites. This exploratory result showed that the most important metabolites for frailty status were LPC16:0, LPC18:2, glycine, valine, and tryptophan (Figures 2C,E); however, univariate analysis showed no significant difference in valine level between the two groups (Table 2).

With regard to the OPLS-DA model of F&S vs. non-frail and non-sarcopenic control, the clustering trend was not sufficiently ideal; a separation trend was only found for the main principal components, and the predictive capacity was low, although this model had certain explainability (R2X = 0.615, R2Y = 0.172, Q2 = 0.032) (Figure 2B). This exploratory model showed that the best discriminant metabolites contributing to the model included a subset of LPC16:0, LPC18:2, valine, and tryptophan (Figures 2D,F). Among these discriminant metabolites, most showed reduced levels in F&S compared with non-frail and non-sarcopenic control; however, the glycine concentration was increased in F&S. These discriminant metabolites in F&S are presented in Table 4; the univariate analysis showed a significant difference in C5 concentration (VIP < 1) between F&S vs. non-frail and non-sarcopenic control, however, no significant differences of valine (VIP > 1) and glycine levels (VIP < 1) were found between the two groups.

**Table 4:** Serum concentrations of discriminant analytes in frailty and sarcopenia (F&S).

**Variables**	**Non-frailty and non-sarcopenia (*n* = 85)**	**Frailty and sarcopenia (*n* = 52)**	**VIP**	**t/Z**	***P*-value**	**FDR**
LPC16:0 (μg/ml)^a^	121.3 (93.21, 147.59)	104.28 (85.27, 129.33)	**3.918**	−2.601	**0.009**	**0.135**
LPC18:2 (μg/ml)^a^	30.46 (24.22, 36.06)	24.36 (17.73, 33.02)	**2.69**	−2.600	**0.009**	**0.091**
valine (μg/ml)	40.69 ± 6.48	39.48 ± 8.92	**1.114**	−1.435	0.151	0.439
Tryptophan (μg/ml)	17.61 ± 2.74	16.36 ± 3.37	**1.006**	−2.553	**0.011**	0.078
Glutamine (μg/ml)	26.13 ± 7.84	25.21 ± 5.24	0.874	−0.126	0.899	0.932
LPC18:1 (μg/ml)	18.20 ± 4.05	17.42 ± 3.93	0.800	−1.200	0.230	0.477
Leucine (μg/ml)	26.06 ± 4.07	25.31 ± 5.43	0.786	−1.124	0.261	0.504
Glutamate (μg/ml)	22.28 ± 3.85	21.10 ± 5.92	0.615	−1.812	0.070	0.338
LPC18:0 (μg/ml)^a^	36.45 (30.29, 39.26)	35.48 (30.24, 43.04)	0.595	0.326	0.744	0.864
Tyrosine (μg/ml)	16.23 ± 2.85	15.80 ± 3.83	0.593	−0.690	0.494	0.753
carnitine (μg/mg)	10.61 ± 2.21	10.05 ± 2.05	0.570	−1.308	0.191	0.461
Ileucine (μg/ml)	12.92 ± 2.52	12.57 ± 3.28	0.493	−1.220	0.222	0.496
glycine (μg/ml)^a^	37.81 (32.42, 48.12)	39.54 (35.36, 43.44)	0.470	0.809	0.418	0.713
C2 (μg/mg)	2.26 ± 0.78	2.12 ± 0.64	0.410	−0.736	0.462	0.744
TMAO (μg/ml) ^a^	0.43 (0.27, 0.59)	0.43 (0.28, 0.7)	0.189	0.333	0.739	0.893
Phenylalanine (μg/ml)	19.25 ± 3.58	19.72 ± 4.15	0.151	0.481	0.630	0.831
C18:1 (μg/mg)^a^	0.10 (0.09, 0.12)	0.09 (0.08, 0.11)	0.066	−1.713	0.087	0.315
C5 (μg/mg)^a^	0.03 (0.02, 0.03)	0.02 (0.02, 0.03)	0.059	−2.995	**0.003**	**0.080**
C3 (μg/mg)	0.11 ± 0.03	0.10 ± 0.04	0.054	−0.581	0.561	0.814
C18 (μg/mg)^a^	0.03 (0.02, 0.03)	0.02 (0.02, 0.03)	0.029	−0.545	0.586	0.809

a *Median and interquartile range (P25, P75)*.

*Bold values mean VIP > 1 statistical significance (p < 0.05), and false discovery rates (FDR) ≤ 0.30. C2, acetylcarnitine; C3, propionyl-L-carnitine; C5, valeryl-L-carnitine; C18, octadecanoyl-L-carnitine; C18:1, octadecenoyl-L-carnitine; LPC16:0, 1-palmitoyl-2-hydroxy-sn-glycero-3-phosphocholine; LPC18:0, 1-stearoyl-2-hydroxy-sn-glycero-3-phosphocholine; LPC18:1, 1-oleoyl-2-hydroxy-sn-glycero-3-phosphocholine; LPC18:2, 1-linoleoyl-2-hydroxy-sn-glycero-3-phosphocholine; TMAO, trimethylamine oxide*.

Permutation tests indicated that the above models were reliable and had a low risk of over-fitting. The area under the curve (AUC) of the above two models were all >0.7 with the discriminatory power to some accuracy (Supplementary Figure 1).

## Discussion

Frailty is associated with a higher risk of disability, hospitalization, and death in older people, playing a central role in poor health later in life. Related biomarkers of frailty can be used in diagnosis and treatment decisions concerning frailty (38, 39). As a reflection of the comprehensive interaction between the genome and environment at the individual level, metabolomics methods are increasingly becoming the preferred approach to uncover frailty- contributing biomarkers and explore the mechanismof frailty (11–13). To our knowledge, the relationship of frailty with AcyCNs and LPCs, as well as amino acids, in older adults has not been well characterized. In the current study, we found that in older men demonstrating the frailty phenotype, amino acid perturbations involved in lower tryptophan and increased glycine levels. With regard to lipid metabolism, the frailty phenotype was characterized by decreased concentrations of C5, LPC16:0, and LPC18:2 and increased levels of C8, C10, C12, and C14. After adjusting for several clinical factors, logistic regression analysis showed that tryptophan, LPC 18:2, LPC16:0, and C5 were negatively associated with frailty whereas C8 and C12 were positively associated with frailty. As an OPLS-DA-based strategy, owing to its ability to handle multiple interdependent variables, we preliminarily identified specific profiles of circulating metabolites LPC16:0, LPC18:2, glycine, and tryptophan that discriminate older men with frailty from non-frailty individuals. Additionally, a combination of serum amino acids and LPCs, specifically LPC16:0, LPC18:2, and tryptophan, were preliminarily identified as characteristic metabotypes in older adults, with an overlap of frailty and sarcopenia in this study. The presence of metabolic differences between older men with and without frailty has the potential to be used as a biomarker for frailty, and overlapped sarcopnia. This may represent a relevant step toward the integration of specific metabolite measurements into the management of frailty and sarcopenia in research and clinical settings.

Specific patterns of circulating amino acids have been associated with frailty; among amino acid profiles, tryptophan breakdown products have been found in many studies. Tryptophan is an essential amino acid that exerts multiple roles in immune, nervous, and muscular system functions (40, 41). Alterations in tryptophan metabolism have been described in the context of low muscle quality, insulin resistance, and frailty in older adults (42). Marron et al. identified metabolites and biological pathways associated with frailty among 287 black men aged 70–81 years from the Health, Aging, and Body Composition (ABC) study. Using LC–MS/MS, they found that lower values of tryptophan, methionine, tyrosine, asparagine, C14:0 sphingomyelin, as well as 1-methylnicotinamide and higher values of glucoronate, N-carbamoyl-beta-alanine, isocitrate, creatinine, C4-OH carnitine, cystathionine, hydroxyphenylacetate, and putrescine were associated with frailty Scale of Aging Vigor in Epidemiology scores in partial Pearson correlation analysis (30). Additional study have demonstrated the occurrence of lower levels of tryptophan, serine, histidine, glycerol, and sphingosine phospholipids and higher levels of hydroxyproline, 3-methylhydropyridine, cystine, and β-aminoisobutyric acid metabolites in variance analysis and linear regression analysis among patients with breast cancer who have frailty (43). Consistent with recent findings, Kameda et al. (44) applied untargeted, comprehensive LC-MS metabolomic analysis to human blood from 19 frail and non-frail elderly patients who were clinically evaluated using the Edmonton Frail Scale. Those authors found that among the 15 frailty markers, tryptophan, proline, isoleucine, leucine, and methionine were reduced. In a population of Spanish older adults recruited from several associations of retired older people, nursing homes, and daycare centers, significant decreases in tryptophan, nitrite, and tyrosine concentrations were noted in frail individuals compared with non-frail persons (45). In the “BIOmarkers associated with Sarcopenia and Physical frailty (PF&S) in EldeRly pErsons” (BIOSPHERE) study, 11 elderly adults aged 70 years and older with PF&S and 10 non-frail and non-sarcopenic controls were included. The results of that study showed that a decline in tryptophan was one of the best predictors for discriminating older people with and without PF&S (46). Our data support those previous studies. It can be deduced that tryptophan, as a radical scavenger, its diminished level could be an important vulnerability for antioxidative defense and might be involved in the pathogenesis of frailty, and overlapped sarcopenia. Notwithstanding, our finding on lower level in tryptophan is not consistent with previous investigation that have reported higher tryptophan concentrations in patients with frailty and type 2 diabetes, according to the European Metabofrail study, which applied a partial least squares-discriminant analysis (PLS-DA)-based analytical strategy to characterize the metabotype (47). Thus, it is essential that further research be performed to confirm the specific role of tryptophan in frailty.

Glycine is a major amino acid and the simplest non-essential amino acid. Recent metabolomics studies have found a relationship between serum levels of glycine and cardiometabolic disease (28, 29). Glycine is involved in anti-inflammatory processes, immune function, and antioxidation reactions (48). In the present study, we observed that the glycine level was higher, after adjusting for clinical factors in logistic regression of Models 2 and 3, and its relationship with frailty did not remain significant, whereas, showed no significant difference in F&S. Valine is a BCAA that has a stimulatory effect on glutamine synthesis and is a major source of nitrogen (49). Although it was found to be involved in distinguishing between the two groups, no significant differences were observed between groups in the serum levels of BCAAs (valine, leucine, and isoleucine) and Glu in the present study. In particular, lower plasma concentrations of the BCAAs leucine and isoleucine have been found in sarcopenic older Norwegian community dwelling adults (50). Additionally, frail older Japanese people showed lower levels of essential amino acids (histidine, isoleucine, leucine, lysine, methionine, phenylalanine, threonine, valine) and tryptophan than their non-frail counterparts (40). Older individuals with frailty and sarcopenia showed higher levels of circulating asparagine, aspartic acid, citrulline, ethanolamine, glutamic acid, sarcosine, and taurine and lower levels of alpha-amino butyric acid and methionine in PLS-DA (46). Notwithstanding, our finding regarding BCAAs is not consistent with previous investigations reporting changes in BCAA concentrations in relation to frailty and sarcopenia. These discrepancies may be owing to differences in clinical characteristics, operational definitions of frailty and experimental designs among studies. Additionally, heterogeneity in dietary and lifestyle habits among participants in the different studies may also contribute to the contrasting results.

LPCs are a major class of glycerophospholipids in human blood. The most abundant LPC in human plasma is LPC 16:0, followed by LPC 18:2, LPC 18:0/18:1, LPC 20:4, and other minor LPC species. LPC has been relevant to muscle mass and mitochondrial oxidative capacity in skeletal muscle (20, 51). Previous studies have demonstrated the occurrence of lower levels of glycerol and sphingosine phospholipids in frail patients with breast cancer (43). In the current study, both frailty individuals and overlapped sarcopenia showed perturbation in the metabolism of LPC16:0 and LPC18:2 in which serum concentrations were lower than those observed in non-frailty individuals. Decreased blood LPC18:2 has previously been shown to be an independent predictor of decline in gait speed, in addition to glucose tolerance, insulin resistance, type 2 diabetes, coronary artery disease, and memory impairment in older adults (19). The homeostasis of phospholipids is achieved by balancing biosynthesis, degradation, recycling, and other processes that add or remove sphingolipids from tissues. The depletion of circulatory phospholipids observed in frailty elderly men could reflect an enhanced demand for lipids to reconstitute cellular membrane turnover. The biological mechanisms by which LPCs play important roles in antioxidation, inflammation and insulin resistance may be important contributors to frailty and sarcopenia (52).

As short-chain AcyCNs, propionyl (C3), methylmalonyl (C4-DC), and isovalerylcarnitine (C5), as well as medium-/long-chain AcyCNs (C6–C20) are produced during fatty acid oxidation (53). The main function of AcyCNs is the transport of long-chain fatty acids to the inner mitochondrial membrane to permit β-oxidation. Other functions of AcyCNs include buffering the mitochondrial acyl-CoA/CoA ratio, oxidation of BCAAs and a protective effect on the endogenous antioxidant defense system (54). Carnitine was found to be correlated with frailty, grip strength, gait speed, or muscle mass (55), but not sarcopenia. In a cross-sectional study of frailty phenotype compiled from older outpatients, the researchers evaluated the carnitine levels in patients with frailty and observed that high levels of carnitine may have a favorable effect on functional status and may be used to treat frailty in older people, however, no specific species of carnitine was detected (56). In this study, we found a lower level of C5 in frailty and overlapped sarcopenia, whereas, higher levels of C8, C10, C12, and C14 in the frailty group; after adjusting for several confounders, C8 and C12 and C5 were independently associated with frailty. In a previous study, C5 was also positively related to SMI (55). Blood level of C5 has been associated with muscle metabolites in frailty. Moreover, C5, Glu, and Gln have been found to be markers of frailty in skeletal muscle (57). Thus, a reduction in C5 level is expected to cause reduced availability of long-chain fatty acids in the mitochondria and an energetic deficit, especially in the most energy-demanding tissues, like skeletal muscle, playing a role in modulating cellular energy production (58). Previous studies have also found higher plasma concentrations of medium- and long-chain AcyCNs, which are correlated with lower levels of physical performance (59) and a greater risk of lower Short Physical Performance Battery score (including balance, gait speed, and 5-repetition sit-to-stand) (17). Similar to the above results, frail participants showed perturbation of acylcarnitine, as shown by the higher levels in medium/long-chain AcyCNs (C8, C10, C12, and C14) in our study. Moreover, Rattray et al. (10) identified 12 significant metabolites, including six carnitines, comprising tetradecanoyl carnitine C14:1, trans-hexadec-2-enoyl-carnitine, linoleyl-carnitine C18:2, vaccenyl-carnitine, stearoyl-carnitine, and hexacosanoyl-carnitine, that could differentiatefrail and non-frail phenotypes, providing evidence that the dysregulation of carnitine shuttle pathways plays a key role in the risk of frailty. The increased level of AcyCNs can indicate mitochondrial dysfunction and a potential increase in the production of reactive oxygen species.

In our study, the significant relationship observed between these frailty-related metabolites indicate that they change in interrelated ways. These metabolites are at the crossroads of multiple biological processes, notebly, mitochondrial function and redox homeostasis besides inflammation and insulin resistance, all of which may contribute to frailty or sarcopenia as per the above discussion. As an intermediate product of fatty acid oxidation, significantly positive correlations were observed among the AcyCNs C8, C10, C12, and C14. The accumulation of these medium-/long-chain AcyCNs may point toward the inability of mitochondria to properly metabolize fatty acids (54, 60). The decline in tryptophan concentration may be related to malnutrition in this study, and a lower C5 concentration showed a positive relationship with tryptophan, possibly because both of these are metabolites are associated with BCAA metabolism (61). C5 was also moderately associated with LPC16:0 and LPC18:2, and the reduction in C5 and LPCs levels may be owing to impaired mitochondrial oxidative capacity (62). The accumulation of non-oxidized fatty acids promotes their conjugation with glycine and carnitine and alternate ways of oxidation (63). The underlying mechanism of the correlations among other metabolites are still unclear, and future research is needed in metabolic pathway analysis.

In the present study, in either univariate or multivariate analysis, the three shared metabolites of frailty and F&S included LPC16:0, LPC18:2 and tryptophan. Two reasons may help to explain the relationship between characteristic metabotypes of frailty and that of F&S. First, at definition level, frailty encompasses several domains (i.e., physical and cognitive), and sarcopenia overlaps with the physical domain of frailty. Gait speed and grip strength are both used to define frailty and sarcopenia, and the above mentioned metabolites are related to these two muscle traits (19). Additionally, specific patterns of circulating tryptophan and LPCs have been associated with muscle mass which is essential in sarcopenia (42, 51); hence, the above metabolites might be more pronounced in metabotype changes with F&S status. Conversely, the existing research findings showed that glycine and some subclasses of AcyCNs are related to frailty (10, 42); however, no studies have reported that these are related to sarcopenia, only related to one or more sarcopenic components (55). Second, from a pathophysiologic point of view, the study of metabolic responses in specific biochemical pathways are of great interest considering the close association between the characteristic metabotypes of frailty and that of F&S. F&S may be considered a prototypical geriatric/geroscience condition, spanning muscle-specific processes to systemic changes (9, 64), even though frailty and sarcopenia are different concepts. We believe that this correlation of metabotypes of frailty with that of F&S emphasizes the interrelation of the above pathophysiological mechanisms.

Body composition, neuroendocrine dysregulation, and immune dysfunction may represent the underlying biologic dynamics that influence the development of frailty in both men and women (65, 66). According to these physiological differences, sex differences in metabolites would be expected, however, the existing evidence is sparse on whether the relationship between metabolic markers and risk of frailty/sarcopenia is the same in men and women. Among previous studies, one study reported that the best multivariate predictive models of of evolution toward pre-frailty include dimethyloxazole, glutamine, and isovalerylcarnitine in men, and dihydroxyphenyl acetic acid, threonine, and mannose in women (58). There are several metabolic studies concerning elderly men. To identify metabolic perturbations that may affect functional decline, non-targeted metabolomics was used in 313 black men in the Health ABC Study, the potential role of amino acid derivatives and products and kidney function early in the development of mobility disability was highlighted (18). Marron et al. identified metabolites associated with frailty among 287 black men, they found that lower values of tryptophan, methionine, tyrosine, asparagine, C14:0 sphingomyelin, etc. were associated with the frailty (30). Murphy et al. (55) performed metabolomics analysis of plasma from 319 black men; the metabolites most strongly correlated with ASM included AcyCN C5 and BCAAs. Of importance, the causes of impaired muscle mass and strength with aging were examined in 119 older men; the results demonstrated a fundamental role of altered mitochondrial metabolism in the pathological losses in sarcopenia among older people (67). In contrast, most previous studies in women have addressed sarcopenic components. A study among community-dwelling older Japanese women found that those with sarcopenia showed lower plasma leucine, BCAA, and essential amino acid concentrations than normal and pre-sarcopenic older women (68) and compounds involved in nitrogen metabolism could be affected in sarcopenia, with an inherent risk in women (51). Importantly, previous metabolic studies have replicated associations between metabolites and phenotypes (55) across populations comprising both men and women, with diverse race/ethnicities and demographics, suggesting some biological variations may have a minimal impact on metabolite relationships. Our study did not include analysis of frail women because of the small sample of female patients who met the inclusion criteria. Gender factor should be considered using reasonable grouping or matching to minimize bias in the future study.

There are some limitations in the present study. First, the study population was relatively small; also, a large number of experimental variables were included in the analyses, which may limit the statistical power. Second, the participants in our study were mostly older men with prefrail status; thus, the findings cannot necessarily be generalized to populations owing to social and cultural differences. Third, other factors may affect circulating metabolites levels, such as lifestyle and dietary habits. Thorough investigations regarding the effect of lifestyle variables, dietary intake, and medication on potential biomarkers should be conducted in further studies to understand all important confounders. Last, this study is a cross-sectional study sought to assess the relationship of metabolites with frailty, and a causal relationship and potential diagnostic biomarkers of metabolic changes in frailty should be investigated in future follow-up studies.

## Conclusion

Our metabolic profiling analysis can provide valuable information regarding the potential biomarkers and possible biological mechanisms in older men. In addition to identifying LPC16:0, LPC18:2, tryptophan, C5, C8 and C12, which appear to be independently correlated with frailty status, we preliminarily demonstrate that the characteristic metabotypes of frailty and that of F&S shared a set of tryptophan, LPC16:0 and LPC18:2, and glycine may specifically discriminate individuals with frailty from non-frailty. The metabolites that were most discriminating of frailty status may imply an underlying mechanism involved in antioxidation and mitochondrial function. These findings offer important references for the management of frailty and sarcopenia. Subsequent studies are needed to confirm our findings and to further determine whether these metabolites can indeed be used as potential biomarkers for frailty.

## Data Availability Statement

The original contributions presented in the study are included in the article/Supplementary Material, further inquiries can be directed to the corresponding authors.

## Ethics Statement

The studies involving human participants were reviewed and approved by Ethics Committee of the Beijing Hospital of the National Health Commission. The patients/participants provided their written informed consent to participate in this study.

## Author Contributions

LM: designed the study, analyzed the data, and drafted the paper. R-yY: performed the study, detection of metabolites, and contributed to the writing of the manuscript preparation. HS: contributed to the design of the work, and helped in execution of research. D-gW: sample collection and preparation, detection of blood chemical parameters. JShi, W-bW, JShe, Y-mD, and G-qF: performed the study and collected the data. P-lY and JD: helped in execution of research. HX: initiated the study, supervised the writing of the manuscript. All authors contributed to the article and approved the submitted version.

## Funding

The study was supported by the grants from the National Key R&D Program of China (No. 2020YFC2009006, No.2020YFC2009000), the National Natural Science Foundation of China (No. 81672075) and Beijing Natural Science Foundation (No.7222156).

## Conflict of Interest

The authors declare that the research was conducted in the absence of any commercial or financial relationships that could be construed as a potential conflict of interest.

## Publisher's Note

All claims expressed in this article are solely those of the authors and do not necessarily represent those of their affiliated organizations, or those of the publisher, the editors and the reviewers. Any product that may be evaluated in this article, or claim that may be made by its manufacturer, is not guaranteed or endorsed by the publisher.
