# Supplementary material for: Specific Metabolites Involved in Antioxidation and Mitochondrial Function Are Correlated With Frailty in Elderly Men
Source: Front Med (Lausanne). 2022 Jan 28;9:816045. doi: 10.3389/fmed.2022.816045 (PMC8833032; doi:10.3389/fmed.2022.816045)
Supplement: Supplementary file 1 [file Data_Sheet_1.docx]

**Suppl. TABLE 1** Logistic regression analysis for associations of serum metabolites with frailty by continuous variables.

| Model | variables | *β* | SE | OR(95%CI) | P value |
| --- | --- | --- | --- | --- | --- |
| MODLE1 | A6 | -0.0964 | 0.0453 | 0.908(0.831,0.992) | **0.0334** |
|  | A9 | 0.0175 | 0.0104 | 1.018(0.997,1.039) | 0.0942 |
|  | C5 | -41.1544 | 15.4761 | <0.001(<0.001,<0.001) | **0.0078** |
|  | C8 | 3.7098 | 4.0191 | 40.844(0.015,>999.999) | 0.356 |
|  | C10 | 2.3957 | 2.6606 | 10.976(0.06,>999.999) | 0.3679 |
|  | C12 | 20.8263 | 11.5274 | >999.999(0.171,>999.999) | 0.0708 |
|  | C14 | 82.7088 | 36.8895 | >999.999(>999.999,>999.999) | **0.025** |
|  | L1 | -0.0105 | 0.00421 | 0.99(0.981,0.998) | **0.0125** |
|  | L4 | -0.0319 | 0.0121 | 0.969(0.946,0.992) | **0.0085** |
| MODLE2 | A6 | -0.0562 | 0.0509 | 0.945(0.856,1.045) | 0.2699 |
|  | A9 | 0.0111 | 0.0113 | 1.011(0.989,1.034) | 0.3259 |
|  | C5 | -40.6207 | 17.1374 | <0.001(<0.001,<0.001) | **0.0178** |
|  | C8 | 2.1202 | 4.1564 | 8.332(0.002,>999.999) | 0.61 |
|  | C10 | 1.0423 | 2.7807 | 2.836(0.012,660.028) | 0.7078 |
|  | C12 | 12.2235 | 12.6589 | >999.999(<0.001,>999.999) | 0.3342 |
|  | C14 | 60.5092 | 40.3777 | >999.999(<0.001,>999.999) | 0.134 |
|  | L1 | -0.0109 | 0.00463 | 0.989(0.98,0.998) | **0.0189** |
|  | L4 | -0.0311 | 0.0135 | 0.969(0.944,0.995) | **0.0212** |
| MODLE3 | A6 | -0.0612 | 0.0524 | 0.941(0.849,1.042) | 0.2423 |
|  | A9 | 0.011 | 0.0117 | 1.011(0.988,1.034) | 0.3484 |
|  | C5 | -46.9208 | 17.807 | <0.001(<0.001,<0.001) | **0.0084** |
|  | C8 | 2.6977 | 4.3693 | 14.846(0.003,>999.999) | 0.5369 |
|  | C10 | 1.685 | 2.9465 | 5.392(0.017,>999.999) | 0.5674 |
|  | C12 | 16.1633 | 13.6027 | >999.999(<0.001,>999.999) | 0.2347 |
|  | C14 | 69.2991 | 42.9713 | >999.999(<0.001,>999.999) | 0.1068 |
|  | L1 | -0.0143 | 0.00493 | 0.986(0.976,0.995) | **0.0038** |
|  | L4 | -0.0437 | 0.0147 | 0.957(0.93,0.985) | **0.003** |

*Note*: Model1: age, body mass index, smoking status; Model2: further controlled for malnutrition, cognitive decline, activities of daily living, polypharmacy, comorbidities; Model3: further controlled for red blood cell and hemoglobin, alanine aminotransferase and uric acid; C5,valeryl-L-carnitine;C8,octanoyl-L-carnitine;C10,decanoyl-L-carnitin;C12,dodecanoy-L-carnitine;C14,tetradecanoyl-L-car-nitine; LPC16:0,1-palmitoyl-2-hydroxy-sn-glycero-3-phosphocholine; LPC18:2,1-linoleoyl-2-hydroxy-sn-glycero-3-phosphocholine.

**Suppl. TABLE 2** Logistic regression analysis for associations of serum metabolites with frailty by categorical variables.

| Variables | Model1 | | | |  | Model 2 | | | |  | Model 3 | | | |
| --- | --- | --- | --- | --- | --- | --- | --- | --- | --- | --- | --- | --- | --- | --- |
|  | *β* | *SE* | OR(95%CI) | *P* |  | *β* | *SE* | OR(95%CI) | *P* |  | *β* | *SE* | OR(95%CI) | *P* |
| Glycine -M3 | 0.663 | 0.380 | 1.941(0.921,4.091) | 0.081 |  | 0.355 | 0.413 | 1.426(0.635,3.203) | 0.390 |  | 0.314 | 0.424 | 1.368(0.596,3.142) | 0.460 |
| -M2 | 0.584 | 0.361 | 1.793(0.883,3.638) | 0.106 |  | 0.380 | 0.386 | 1.462(0.685,3.118) | 0.326 |  | 0.324 | 0.398 | 1.382(0.634,3.015) | 0.416 |
| -M1 | 0.827 | 0.373 | 2.286(1.101,4.747) | **0.027** |  | 0.613 | 0.397 | 1.846(0.847,4.022) | 0.123 |  | 0.566 | 0.409 | 1.76(0.789,3.927) | 0.170 |
| C5-M3 | -0.777 | 0.409 | 0.46(0.206,1.024) | 0.057 |  | -0.659 | 0.452 | 0.518(0.214,1.254) | 0.145 |  | -0.841 | 0.470 | 0.431(0.172,1.083) | 0.073 |
| -M2 | -0.568 | 0.403 | 0.567(0.257,1.249) | 0.159 |  | -0.292 | 0.441 | 0.747(0.314,1.772) | 0.507 |  | -0.453 | 0.459 | 0.636(0.258,1.564) | 0.324 |
| -M1 | 0.021 | 0.410 | 1.021(0.457,2.283) | 0.959 |  | 0.133 | 0.449 | 1.142(0.474,2.755) | 0.767 |  | 0.023 | 0.463 | 1.023(0.413,2.536) | 0.961 |
| C10-M3 | 0.795 | 0.389 | 2.213(1.033,4.742) | **0.041** |  | 0.648 | 0.423 | 1.911(0.834,4.38) | 0.126 |  | 0.741 | 0.442 | 2.097(0.882,4.986) | 0.094 |
| -M2 | 0.628 | 0.368 | 1.875(0.912,3.854) | 0.088 |  | 0.507 | 0.405 | 1.661(0.750,3.676) | 0.211 |  | 0.567 | 0.417 | 1.762(0.778,3.989) | 0.174 |
| -M1 | 0.803 | 0.375 | 2.232(1.070,4.653) | **0.032** |  | 0.744 | 0.400 | 2.105(0.962,4.608) | 0.063 |  | 0.738 | 0.408 | 2.091(0.939,4.655) | 0.071 |
| C14-M3 | 0.873 | 0.382 | 2.394(1.132,5.064) | **0.022** |  | 0.695 | 0.415 | 2.003(0.888,4.517) | 0.094 |  | 0.784 | 0.432 | 2.19(0.939,5.108) | 0.070 |
| -M2 | 0.397 | 0.356 | 1.487(0.739,2.99) | 0.266 |  | 0.222 | 0.387 | 1.249(0.585,2.667) | 0.566 |  | 0.380 | 0.398 | 1.462(0.670,3.190) | 0.340 |
| -M1 | 0.203 | 0.389 | 1.225(0.572,2.624) | 0.601 |  | 0.197 | 0.423 | 1.218(0.532,2.79) | 0.641 |  | 0.220 | 0.432 | 1.246(0.534,2.908) | 0.610 |
|  |  |  |  |  |  |  |  |  |  |  |  |  |  |  |

*Note*: Model1: age, body mass index, smoking status; Model2: further controlled for malnutrition, cognitive decline, activities of daily living, polypharmacy, comorbidities; Model3: further controlled for red blood cell and hemoglobin, alanine aminotransferase and uric acid; Measured metabolites were used as categorical variables: lower quartile (M1), median (M2), upper quartile (M3) versus lowest quartile.C5,valeryl-L-carnitine; C10,decanoyl-L-carnitin; C14,tetradecanoyl-L-car-nitine

**Suppl. Figure1**


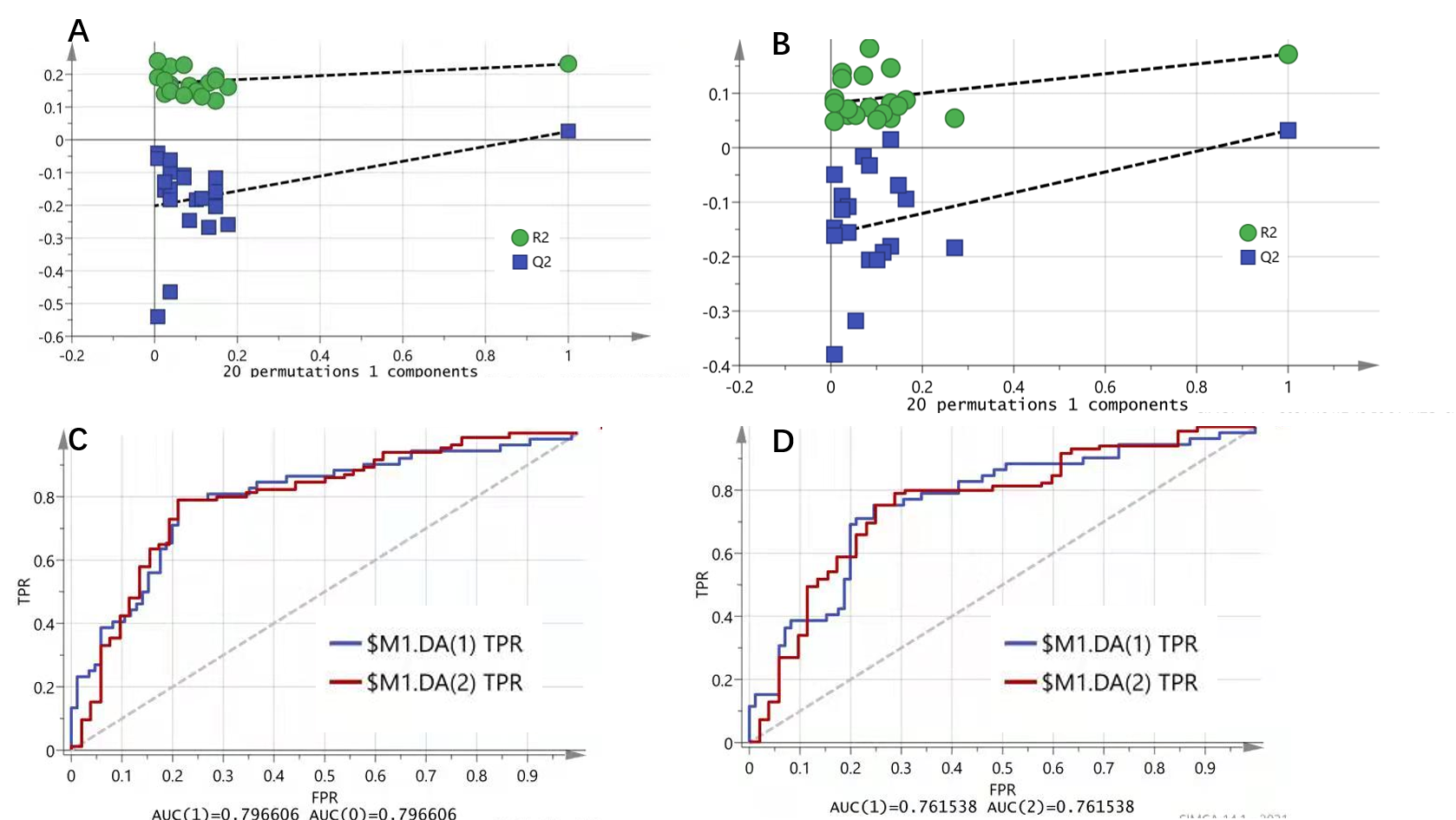


**FIGURE 1** Validation of OPLS-DA analysis. **(A)** Permutation tests of OPLS-DA model analysis for frailty versus nonfrailty groups. **(B)** Permutation tests of OPLS-DA model analysis for frailty and sarcopenia (F&S) versus nonfrail and nonsarcopenic control. **(C)** The area under the curve (AUC) for separation of frailty and nonfrailty groups. **(D)** The area under the curve (AUC) for separation of frailty and sarcopenia (F&S) versus nonfrail and nonsarcopenic control.
